# Circulating microRNA Signature Associated to Interstitial Lung Abnormalities in Respiratory Asymptomatic Subjects

**DOI:** 10.3390/cells9061556

**Published:** 2020-06-26

**Authors:** Blanca Ortiz-Quintero, Ivette Buendía-Roldán, Eric Gustavo Ramírez-Salazar, Yalbi I Balderas-Martínez, Sandra Lizbeth Ramírez-Rodríguez, Karen Martínez-Espinosa, Moisés Selman

**Affiliations:** 1 Unidad de Investigación, Instituto Nacional de Enfermedades Respiratorias Ismael Cosío Villegas. Calzada de Tlalpan 4502, Colonia Sección XVI, Mexico City 14080, Mexico; ivettebu@yahoo.com.mx (I.B.-R.); yalbibalderas@gmail.com (Y.IB.-M.); sandraliz2610@gmail.com (S.L.R.-R.); karen.mtz92@gmail.com (K.M.-E.); 2 Laboratorio de Genómica del Metabolismo Óseo, Instituto Nacional de Medicina Genómica, Periférico Sur 4809, Arenal Tepepan, Tlalpan, Mexico City 14610, Mexico; eramirez@inmegen.edu.mx

**Keywords:** interstitial lung abnormalities (ILA), circulating microRNAs, biomarkers, lung fibrosis

## Abstract

Interstitial lung abnormalities (ILA) are observed in around 9% of older respiratory asymptomatic subjects, mainly smokers. Evidence suggests that ILA may precede the development of interstitial lung diseases and may evolve to progressive fibrosis. Identifying biomarkers of this subclinical status is relevant for early diagnosis and to predict outcome. We aimed to identify circulating microRNAs (miRNAs) associated to ILA in a cohort of respiratory asymptomatic subjects older than 60 years. We identified 81 subjects with ILA from our Lung-Aging Program in Mexico City (*n* = 826). We randomly selected 112 subjects without ILA (Ctrl) from the same cohort. Using polymerase chain reaction PCR-Array technology (24 ILA and 24 Ctrl, screening cohort) and reverse-transcriptase quantitative polymerase chain reaction (RT-qPCR) (57 ILA and 88 Ctr, independent validation cohort) we identified seven up-regulated miRNAs in serum of ILA compared to Ctrl (miR-193a-5p, *p* < 0.0001; miR-502-3p, *p* < 0.0001; miR-200c-3p, *p* = 0.003; miR-16-5p, *p* = 0.003; miR-21-5p, *p* = 0.002; miR-126-3p, *p* = 0.004 and miR-34a-5p, *p* < 0.005). Pathways regulated by these miRNAs include transforming growth factor beta (TGF-β), Wnt, mammalian target of rapamycin (mTOR), Insulin, mitogen-activated protein kinase (MAPK) signaling, and senescence. Receiver operator characteristic (ROC) curve analysis indicated that miR-193a-5p (area under the curve AUC: 0.75) and miR-502-3p (AUC 0.71) have acceptable diagnostic value. This is the first identification of circulating miRNAs associated to ILA in respiratory asymptomatic subjects, providing potential non-invasive biomarkers and molecular targets to better understand the pathogenic mechanisms associated to ILA.

## 1. Introduction

Interstitial lung abnormalities (ILA) are defined as specific patterns of increased lung densities identified on high-resolution computed tomography (HRCT) scans, usually in subjects without previous diagnosis of pulmonary disease. ILA are frequently observed with advanced age and are associated with smoking history and environmental pollution [1,2,3]. However, ILA are also observed in non-smokers and in some studies the development of these abnormalities was not dependent on smoking status at baseline [4]. In addition, subjects with ILA show a higher frequency of the gain-of-function *MUC5B* common variant gene [5,6,7]. More recently, a genome-wide association study (GWAS) performed in individuals with ILA from six different cohorts, confirmed this association and described novel genome-wide associations near *IPO11* (rs6886640), *FCF1P3* (rs73199442), and near *HTR1E* (rs7744971) gene [8].

Importantly, ILA are associated with reduced lung function and exercise capacity, and increased risk of all-cause and respiratory mortality [1,9,10,11]. Furthermore, several recent studies linked ILA to increased risk for progressive pulmonary fibrosis, lung cancer and lung cancer-associated mortality [12].

Nevertheless, the mechanisms associated to ILA development and putative progression are unknown. In this context, identifying molecular biomarkers of this subclinical disease status is relevant for early diagnosis, to predict outcome, and to reveal some of the mechanisms likely involved.

MicroRNAs (miRNAs) are non-coding RNA that regulate gene expression by blocking the translation of target messenger RNA [13,14]. They have emerged as potential regulators of pathogenic mechanisms and biomarkers for several diseases including respiratory diseases [15,16]. Moreover, miRNAs regulate pathways involved in major mechanisms as inflammation, cellular senescence, and apoptosis, which are associated to the pathogenesis of several age-related respiratory diseases such as idiopathic pulmonary fibrosis (IPF) and lung cancer [17,18,19,20,21,22]. In particular, miRNAs secreted into blood circulation (circulating miRNAs) are useful because they have high stability, disease-specific expression patterns and are easy to detect and quantify [23,24]. The pathophysiological involvement of circulating miRNAs in age-related respiratory diseases is still an ongoing research area, which includes investigating their value as non-invasive biomarkers [25,26,27,28,29]. However, to date, there are no studies approaching miRNA expression in subjects with the subclinical presence of ILA. In this context, we aimed to identify circulating miRNAs and associated pathways in a cohort of respiratory asymptomatic subjects with ILA.

## 2. Materials and Methods

### 2.1. Study Population

From March 2015 to July 2019, 860 respiratory asymptomatic volunteers aged 60 or older were recruited in the “Lung Aging Program” of our Institute. In this study, we identified 81 subjects from this cohort that presented interstitial lung abnormalities (ILA) defined as asymptomatic respiratory individuals that showed by high resolution computed tomography (HRCT) some or a combination of the following images: ground glass attenuation, diffuse nodules, (both associated to inflammatory ILA), reticular opacities, honeycombing or traction bronchiectasis (associated to fibrotic ILA) affecting more than 5% of nondependent lung involvement. Thus, 112 individuals from the same cohort with normal HRCT were randomized selected as controls (Ctrl). For this study, we randomly divided the participants in two cohorts: a screening cohort consisting of 24 ILA and 24 Ctrl; and a second validation cohort consisting of 57 ILA and 88 Ctrl.

At the first visit, in addition to the HRCT, we applied questionnaires for demographics data, performed pulmonary function tests, and obtained blood samples.

All participants signed a consent letter, and the Scientific and Ethic Committee of Instituto Nacional de Enfermedades Respiratorias Ismael Cosio Villegas, Mexico, approved this study (C76-17).

### 2.2. Study Design

For the screening phase, we analyzed the serum miRNA expression profile of four pools (six samples per pool) of each study group using the TaqMan Human MicroRNA Array Panel v2.0, which includes probes for 377 human miRNAs (see details in Section 2.5). This approach allowed the profiling of 377 miRNAs in 24 ILA and 24 Ctrl samples (screening cohort). This screening method was chosen to find robust differences between the study groups. We identified the differentially expressed miRNAs in ILA compared to Ctrl (*p* < 0.05 and log_2_Fold Change > 1.4) and further validated the candidate miRNAs with reverse-transcriptase quantitative polymerase chain reactions (RT-qPCR) using individual samples from the independent validation cohort consisting of 57 ILA and 88 Ctrl. Additionally, we tested in the validation cohort, five selected miRNAs previously associated to senescence and IPF (miR-34a-5p, miR-126-3p, miR-30b-5p, miR-21-5p and miR-106-5p) in literature [16,18,22,29,30,31]. We added these relevant miRNAs because ILA has been linked to increased risk for pulmonary fibrosis and senescence-related disease, and some candidate miRNAs may not be detected in our broad screening phase. We performed in silico analysis of experimentally validated targets of the candidate miRNAs and an enriched pathway analysis using Kyoto Encyclopedia of Genes and Genomes (KEGG) database and bioinformatic tools (see details in Section 2.7) in order to investigate whether the potential ILA-associated miRNAs correlate with pathophysiologic mechanisms. We also investigated the potential diagnostic value of candidate miRNAs using receiver operator characteristic (ROC) curves. In addition, correlation analysis was evaluated between candidate miRNAs and clinical data. Supplementary Figure S1 shows a workflow graph of the study design.

### 2.3. Serum Samples

Blood samples were drawn from the cubital vein and collected into BD Vacutainer SST blood collection tubes without anticoagulant and with a clot separator gel (Becton and Dickinson, REF 368159). After 30 min, serum was separated by centrifugation at 1200× *g* for 15 min (room temperature), aliquoted and stored at −80 °C until use.

### 2.4. RNA Isolation from Serum

Extraction of RNA from serum samples (200 μL) was performed using the miRNeasy Serum/Plasma Kit (cat. 217184, QIAGEN, Hilden, Germany) according to the manufacturer’s instructions. Synthetic *Caenorhabditis elegans* (*C. elegans*) miRNA cel-miR-39 was added to serum samples during the extraction step (validation cohort). This enables normalization for any nonspecific losses incurred during miRNA purification [32].The RNA concentration and purity were assessed using a Nanodrop ND-2000 spectrophotometer (Thermo Fisher Scientific, Waltham, MA, USA). RNA samples were stored at −80 °C until use. We assessed hemolysis in all serum samples by measuring the absorbance of hemoglobin at 414 nm with a Nanodrop ND-2000 spectrophotometer (Thermo Fisher Scientific, Waltham, MA, USA), and later in 50% randomly selected RNA samples by measuring the delta Ct value of miR-23a and miR-451 by RT-qPCR [33].

### 2.5. MicroRNA PCR Arrays

For the screening phase, we analyzed the miRNA expression profile of four RNA pools (six samples per pool) of each study group using TaqMan Human MicroRNA Array Panel v2.0 (Applied Biosystems, CA, USA), which included Card A in a 384-well format and probes for 377 human miRNAs. This approach allowed the profiling of 377 miRNAs in 24 samples of ILA and 24 samples of Ctrl (screening cohort). Quantification procedure was performed according to the manufacturer’s instructions. Briefly, we combined the RNA (from individual serum samples) to make four ILA pools and four Ctrl pools. Each pool contained RNA from 6 individual samples. Following this, 30 ng of pooled RNA was reverse transcribed using the Megaplex RT stem-loop primer pool A (Applied Biosystems, Foster City, CA, USA). Subsequently, Megaplex RT products were pre-amplified using Megaplex PreAmp Primers (Pool A) and TaqMan PreAmp Master Mix (Applied Biosystems). Real-time PCR reactions were performed on the 7900 HT (Applied Biosystems) with the recommended cycling conditions. The differential expression of miRNAs between study groups was assessed using the Expression Suite Software v1.0.3 (Life Technologies). Data were normalized using the mean of the expression value (Ct) of all expressed miRNAs on the plate [34]. Only Raw Ct values lower than 36 were considered for analysis. The levels of miRNAs that showed a *p* < 0.05 and a log_2_ foldchange > 1.4 in ILA compared to Ctrl were considered differentially expressed.

### 2.6. Validation by RT-qPCR

For the validation phase, the levels of seven candidate miRNAs (identified in the screening phase) were quantified in individual serum samples of 57 ILA and 88 Ctrl (validation cohort), by TaqMan Advanced miRNA Assays (Thermo Fisher Scientific). Briefly, total RNA was reverse transcribed using a TaqMan^®^ Advanced miRNA cDNA synthesis kit (Thermo Fisher Scientific), according to the manufacturer’s instructions. Then miRNAs were amplified by qPCR using TaqMan Advanced miRNA Assays for miR-532-5p (Assay ID, 478151_mir), miR-193a-5p (Assay ID, 477954_mir), miR-16-5p (Assay ID, 477860_mir), miR-9-5p (Assay ID, 478214_mir), miR-502-3p (Assay ID, 478348_mir), miR-200c-3p (Assay ID, 478351_mir), miR-95-3p (Assay ID, 478213_mir). Additionally, we tested the levels of five selected miRNAs associated to senescence and idiopathic pulmonary fibrosis (IPF) from literature: miR-34a-5p (Assay ID, 478048_mir), miR-126-3p (Assay ID, 477887_mir), miR-30b-5p (Assay ID, 478007_mir), miR-106b-5p (Assay ID, 478412_mir), and miR-21-5p (Assay ID, 477975_mir). qPCR was performed on a Step-One-Plus Real-Time PCR System (Applied Biosystems). All reactions were performed in triplicate. qPCR data were normalized using the mean of the exogenous spike-in miRNA cel-miR-39-3p (Assay ID 478293_mir) and the mean of the endogenous reference miRNA miR-511-5p (Assay ID 478970_mir) which was previously selected as the most stable normalizer by the NormFinder software [35]. The 2^−ΔCt^ method was used to calculate the relative expression quantities (RQ) of miRNAs levels. RQ = 2^−ΔCt^, ΔCt = Average Mean Ct _miRNA_ – Average Mean Ct _normalizers_.

### 2.7. MiRNA Target Genes and Pathways Enrichment Analysis

Experimentally validated target genes of the candidate miRNAs were obtained through miRNet (http://mirnet.ca). miRNet is a platform that collect miRNAs-target interaction experimentally validated from different databases (miRTarBase, TarBase, miRecords) [36]. We performed an enrichment pathway analysis using Rstudio version 1.2.5033 (https://www.rstudio.com) with R version 3.6.2 (https://www.R-project.org/), and the enrichR package version 2.1 (https://CRAN.R-project.org/package=enrichR), and KEGG_2019_Human database. Pathways with a significant enrichment has an adjusted *p* < 0.05, except for miRNAs miR-193a-5p and miR-502-3p (non-adjusted *p* < 0.05). We used ggplot2 package to generate the graphs to visualize the pathway enrichment analysis (https://ggplot2.tidyverse.org). Networks were built to summarize the results using Cytoscape version 3.8.0 [37].

### 2.8. Statistical Analysis

Descriptive data are presented as frequency tables, mean and standard deviation (SD). Univariate analyses of baseline characteristics were performed with a t-test or chi-squared test as appropriate for the data. We performed a normality test with Kolmogorov-Smirnov. The differences of miRNAs expression levels among the study groups were analyzed by a Mann–Whitney U test. Values of *p* < 0.05 were considered significant. Correlations were evaluated between candidate miRNAs and clinical data using Spearman test. Binary logistic regression was used in receiver operator characteristic (ROC) curve analysis. Analysis were performed using STATA version 15.0 (StataCorp, College Station, Texas, USA) and IBM SPSS Statistics for Windows, version 24.0 (Armonk, NY: IBM Corp, NY, USA). Graphs were constructed using GraphPad Prism version 8.

## 3. Results

### 3.1. Study Population

Clinical and demographic characteristics of ILA and Ctrl from the validation cohort are shown in Table 1 and from the screening ILA and Ctrl groups in Supplementary Table S1. ILA were older (*p* < 0.0001), with male predominance (*p* = 0.01) and high frequency of diabetes (*p* = 0.02) than Ctrl in the validation cohort. As expected, ILA subjects showed lower oxygen saturation (spO2) at rest (*p* = 0.03), and after exercise (*p* = 0.0004), and less walked meters in the 6 min test (6MWT) (*p* = 0.04). They also showed reduced diffusion capacity of the lung for carbon monoxide (DLCO) adjusted (*p* < 0.0001) and DLCO/VA (alveolar volume) (*p* = 0.001) compared to Ctrl. There were no significant differences in the body mass index (BMI), smoking history and diagnosis of systemic hypertension and gastrointestinal reflux between the two study groups.

### 3.2. Identification of Circulating miRNAs Differentially Expressed in ILA Subjects

**Figure 1:**
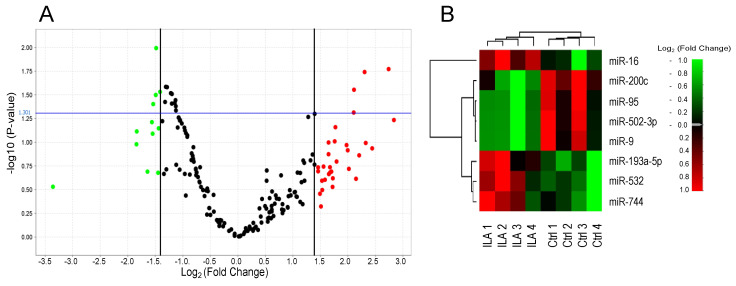
Identification of microRNAs differentially expressed in ILA subjects in the screening cohort. Volcano plot (**A**) and heat map (**B**) of differentially expressed miRNAs in four pools of six samples of ILA compared to four pools of six samples of Ctrl (*p* < 0.05 and log_2_ fold change > 1.4) using PCR Array. In the volcano plot, significant miRNAs are indicated in red (up-regulated) or green (down-regulated) above the cutoff *p*-value (blue line, *p* < 0.05). ILA: subjects with interstitial lung abnormalities. Ctrl: control group.

The miRNA expression profiling of ILA and Ctrl from the screening cohort allowed the identification of four upregulated miRNAs (miR-532-5p, miR-193a-5p, miR-16-5p and miR-744-5p) and four downregulated miRNAs (miR-9-5p, miR-502-3p, miR-200c-3p and miR-95-3p) in ILA compared to Ctrl (*p* < 0.05; log_2_ Fold Change > 1.4) (Figure 1 and Supplementary Table S2).

We further tested the levels of seven of these candidate miRNAs in individual serum samples using an independent validation cohort of 57 ILA patients and 88 Ctrl, by RT-qPCR. We did not test the miR-744-5p because its p-value was too close to 0.05 (*p* = 0.049, Supplementary Table S2). Additionally, we examined the levels of five selected miRNAs (miR-34a-5p, miR-126-3p, miR-30b-5p, miR-106b-5p, and miR-21-5p) associated to senescence and IPF [16,18,22,29,30,31] in literature.

**Figure 2:**
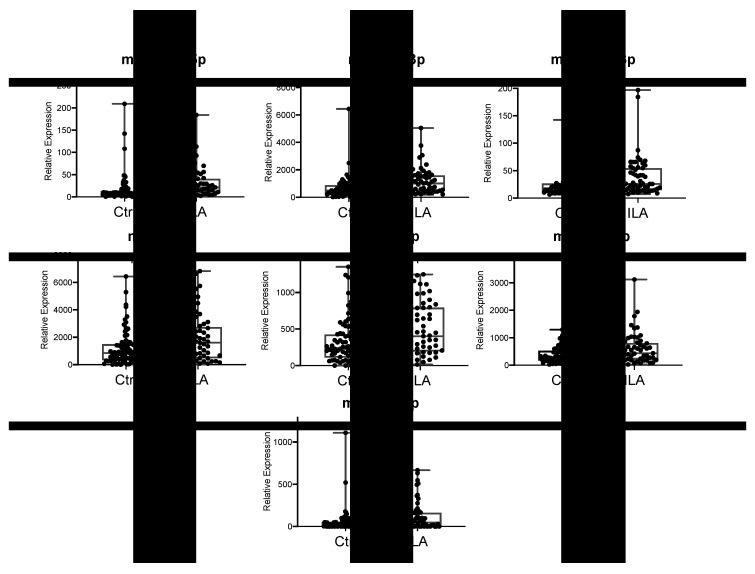
Validation of candidate miRNAs differentially expressed in ILA subjects. Graphs show the quantitative real-time PCR analysis of differentially expressed miRNAs in serum of ILA (*n* = 57) compared to Ctrl (*n* = 88) from the validation cohort. ILA: subjects with interstitial lung abnormalities. Ctrl: control group.

Results obtained in this validation cohort showed that relative expression levels of seven miR-(193a-5p, miR-502-3p, miR-200c-3p, miR-16-5p, miR-21-5p, miR-126-3p and miR-34a-5p) were significantly higher (*p* < 0.05) in ILA compared to Ctrl (Figure 2). Among them, four miRNAs were candidates discovered in the screening phase (miR-193a-5p, miR-502-3p, miR-200c-3p, miR-16-5p), and three miRNAs were the additional candidates selected from literature as associated to IPF (miR-21-5p, miR-126-3p and miR-34a-5p). Meanwhile, relative expression levels of miR-9-5p, miR-95 and miR-532 (from the screening cohort), and miR-30b-5p and miR-106b-5p (additional miRNAs from literature) were not statistically different among study groups (data not shown). Correlation analysis indicated that gender, age, smoking history, BMI or lung functional parameters did not correlate with levels of any candidate miRNA (Supplementary Table S4). We found that some miRNAs correlated with some respiratory functional parameters related to gas exchange such as DLCO and oxygen saturation, but their correlation values were too weak (*r* < 0.2) (Table S4).

Therefore, these results revealed seven candidate miRNAs whose circulating levels are associated to ILA in asymptomatic subjects in a relatively large validation cohort.

By contrast, miR-502-3p and miR-200c-3p that were downregulated in the screening testing were found upregulated in the validation. The discrepancy observed may be due to intrinsic limitation of our screening method (use of pooled samples as well as limited number of samples). We consider that RT-qPCR results are more reliable because individual and higher number of samples were analysed in an independent cohort of 57 ILA and 88 Ctrl, then were confirmed as upregulated the miRNAs levels.

Interestingly, when we analyzed the miRNA levels among the ILA patients in relation with their HRCT predominant pattern we found significant lower levels of miR-16-5p in ILA with reticular opacities, compared to ground glass attenuation (*p* = 0.04) (supplementary Figure S1). This observation suggests that miR-16-5p may be a biomarker for distinguishing between fibrotic ILA (reticular pattern) vs. nonfibrotic ILA (ground glass attenuation) in aging lungs.

### 3.3. ILA-Associated miRNAs Correlate with Pathophysiologic Mechanisms of Chronic Lung Diseases

**Figure 3:**
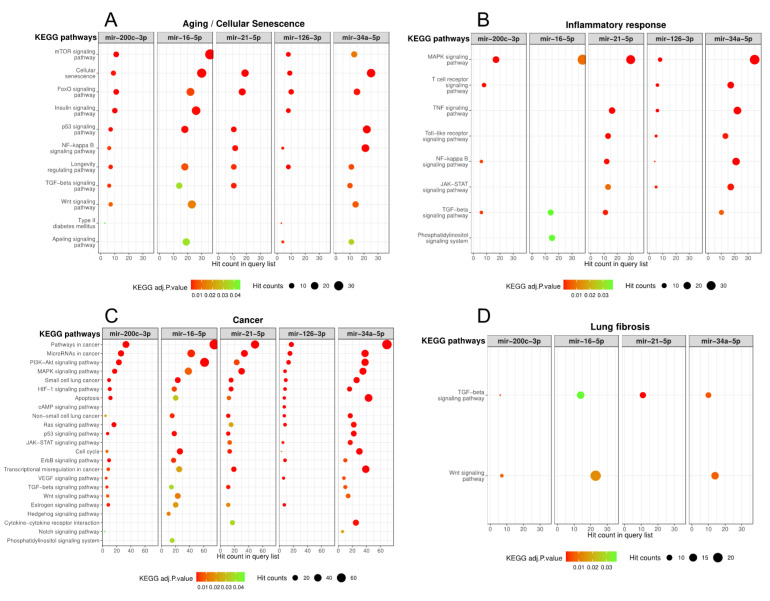
Significantly enriched pathways of ILA-associated miRNAs involved in (**A**) aging/cellular senescence, (**B**) inflammatory response, (**C**) cancer and (**D**) lung fibrosis. Figure shows the KEGG enriched pathways (adjusted *p* < 0.05) using the experimentally validated targets of ILA-associated miRNAs. Colors indicate p-values and circle sizes indicate number of target genes in each pathway. KEGG: Kyoto Encyclopedia of Genes and Genomes database.

In order to investigate whether the increased levels of miRNAs in ILA correlate with pathophysiology of chronic respiratory diseases, we performed in silico analysis of experimentally validated targets of the seven candidate miRNAs and an enriched pathway analysis using KEGG database. We obtained the significantly enriched pathways for each miRNA´s target genes using an adjusted *p* < 0.05 and a non-adjusted *p* < 0.05 for miR-193a-5p and miR-502-3p because the experimentally validated target genes for these two miRNAs were limited (Supplementary Table S3). Among the identified enriched pathways, we further selected those that are involved in aging/cellular senescence, inflammatory response, cancer and lung fibrosis, because these major biological processes are associated to chronic respiratory diseases and ILA-related diseases. Figure 3 shows that miR-200c-3p, miR-16-5p, miR-21-5p, miR-126-3p and miR-34-5p were strongly associated to several pathways related to aging/senescence, inflammatory response, cancer and lung fibrosis (Figure 3A–D). In particular, transforming growth factor beta (TGF-β) signaling pathway (miR-200c-3p, miR-16-5p, miR-21-5p and miR-34-5p) and Wnt signaling pathway (miR-200c-3p, miR-16-5p and miR-34-5p) are known pathways directly associated to IPF and lung fibrosis in literature [38,39].

**Figure 4:**
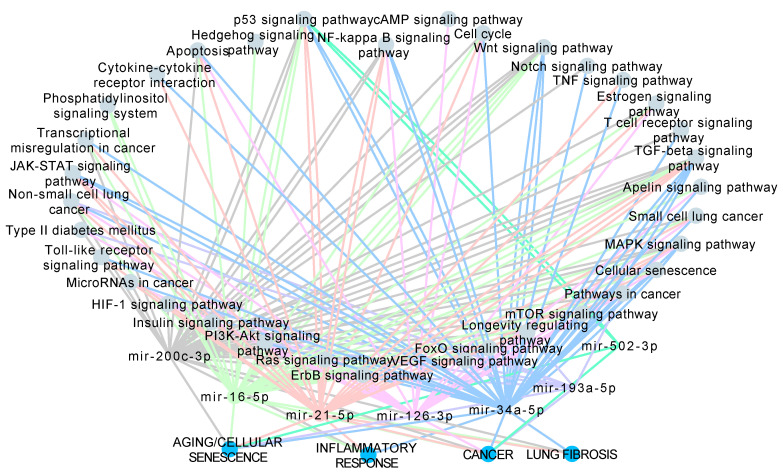
Network of the ILA-associated miRNAs and their enriched pathways involved in the biological processes (**A**) aging/cellular senescence, (**B**) inflammatory response, (**C**) cancer and (**D**) lung fibrosis. Nodes represent either pathways, miRNAs, or biological processes. Edge colors are specific for each miRNA.

In contrast, miR-193a-5p and miR-502-3p had limited information regarding experimentally validated targets and enrichment pathways in KEGG database (Supplementary Table S3 and Supplementary Figure S2). However, we found some significantly enriched pathways associated to cancer and aging/cellular senescence such as erythroblastic leukemia viral oncogene homologue (ErbB) signaling pathway, hypoxia Inducible Factor 1 (HIF-1) signaling pathway, non-small cell lung cancer (NSCLC), pathways in cancer, phosphoinositide 3 kinase-protein kinase B (PI3K-Akt) signaling pathway and longevity regulation pathway for miR-193a-3p; while p53 signaling pathway was enriched for miR-502-3p putative target genes (Supplementary Table S3 and Supplementary Figure S2). To visualize these results, we constructed a network of the ILA-associated miRNAs and their selected KEGG enriched pathways (Figure 4). The network is composed of 35 pathways that were classified into four categories: Aging/Cellular Senescence (12 pathways), Inflammatory Response (9 pathways), Cancer (25 pathways), and Lung Fibrosis (4 pathways). PI3K-Akt signaling pathway, HIF-1 signaling pathway, Longevity regulating pathway, Non-small cell lung cancer, and Pathways in cancer are shared by six miRNAs while Small lung cancer, Cell cycle, and Cellular senescence are shared by five miRNAs (Figure 4).

### 3.4. Serum miRNAs as Biomarkers for Discriminating the Presence or Absence of ILA in Asymptomatic Subjects

**Figure 5:**
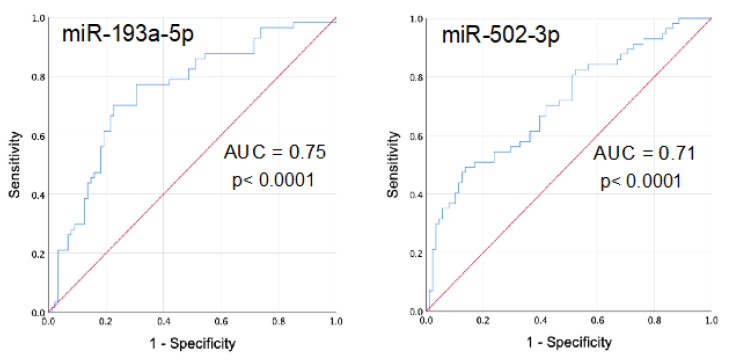
Receiver operator characteristic (ROC) curve analysis of circulating miR-193a-5p and miR-502-3p among ILA and Ctrl. Graphs show the ROC curve of miR-193a-5p and miR-502-3p as a single biomarker for predicting the presence of interstitial lung abnormalities in asymptomatic subjects. AUC: area under the curve. ILA: subjects with interstitial lung abnormalities. Ctrl: control group.

To investigate a potential diagnostic value of the differentially expressed miRNAs, we performed a receiver operator characteristic (ROC) curve analysis for the seven upregulated miRNAs in ILA. Results indicated that levels of miR-193a-5p and miR-502-3p discriminate ILA from Ctrl with an area under the curve (AUC) of 0.75 and 0.71, respectively (Figure 5). The rest of the miRNAs showed AUC values below 0.70 (from 0.65 to 0.66, data not shown). Data of the diagnostic accuracy of miR-193a-5p and miR-502-3p are shown in Table 2. We found that miR-193a-5p had the best sensitivity (61%) at a fixed specificity of 81% as a single biomarker compared to miR-502-3p (sensitivity 51%). Additionally, we performed logistic regression analysis to combine these two candidate miRNAs in order to improve the diagnostic utility. As it is shown in Table 2, sensitivity values did not improve by combining miR-193a-5p and miR-502-3p.

## 4. Discussion

Recent studies performed in large cohorts, including healthy elderly patients, have revealed the presence of interstitial lung abnormalities [1,8,9,10,40]. ILA can be observed in adults without symptoms (subclinical as in our study) but may represent in some cases an early diagnosis of an inflammatory interstitial lung disease, or an epithelial-driven fibrosis such as IPF [41].

Importantly, at diagnosis ILA may be fibrotic characterized by predominance of reticulation and honeycombing on HRCT, or non-fibrotic (usually ground-glass opacities) [4].

Longitudinal studies have demonstrated that while in most of individuals these abnormalities persist without progression, in 20–40% of the cases imaging abnormalities are progressive which is usually associated to an increased decline in pulmonary function and increased rate of mortality [1,8,42]. Moreover, in a recent 5-years longitudinal study, that included a high-risk population of asymptomatic first-degree relatives of patients with familial interstitial pneumonia, it was found that the majority of individuals with early/mild ILA at the time of enrollment had evidence of progression to an interstitial lung disease [43]. All these studies support the notion that screening for ILA might eventually provide a tool for the early identification of an ILD, including IPF which is the most aggressive of them [44,45].

However, ILA represent a complex and still poorly characterized disorder, and the mechanisms implicated in its development and progression are unclear. In this setting, the putative role of miRNA dysregulation in its pathogenesis as well as the potential as diagnostic biomarkers had not been explored.

Our study revealed by the first time that several miRNAs were upregulated in subjects with ILA suggesting the putative dysfunction of some biologically meaningful pathways. Outstandingly, miR-200c-3p, miR-16-5p, and miR-34-5p are involved in two critical profibrotic pathways, TGF-β and Wnt signaling that have been implicated in IPF and other fibrotic lung disorders [33,34]. TGF-β, is a pleiotropic factor that regulates a variety of relevant biological processes, and during a fibrotic response, TGF-β enhances the differentiation of fibroblast to myofibroblast, and induces the over-expression of extracellular matrix molecules, leading to irreversible structural alterations and tissue stiffening. TGF-β also stimulates the production of a number of profibrotic growth factors such as connective tissue growth factor and platelet-derived growth factor [41]. Interestingly, we also found the upregulation of miR-21 which is increased in experimental lung fibrosis as well as in the lungs of IPF patients where it localizes to myofibroblasts. Moreover, Yang et al. [30] reported that miR-21 and miR-200c were significantly increased in serum of slowly progressive IPF patients compared to healthy controls, while miR-21 was also upregulated in rapid progressive IPF patients.

In a bidirectional relationship, TGF-β1 induced miR-21 expression while miR-21 in turn promoted TGF-β1-induced profibrotic phenotype of lung fibroblasts [16,29].

It is important to emphasize that in bleomycin-induced lung fibrosis, miR-21 is elevated during the inflammatory and fibrotic phases suggesting that it may contribute to both, inflammation and fibrosis [29].

Actually, in our study, the network constructed with the KEGG enriched pathways revealed the dysregulation of nine pathways related to the inflammatory response, and four from lung fibrosis.

On the other hand, aberrant Wnt/β-catenin signaling activity has also been shown in human and experimental pulmonary fibrosis [46,47], promoting in fibroblasts a pro-fibrotic phenotype enhancing migration, proliferation and excessive production of extracellular matrix components such as fibrillar collagens [48,49].

Moreover, it was recently demonstrated that chronic Wnt/β-catenin signaling contributes to alveolar epithelial cell senescence and reprogramming, a process that is strongly involved in a profibrotic response [49]. Cellular senescence is a stress response that leads to permanent cell cycle arrest and a complex proinflammatory and matrix-degrading molecules secretory phenotype [44]. Persistent epithelial cell senescence has been demonstrated in IPF and experimental lung fibrosis models where could induce the activation of lung fibroblasts via expression of growth factors [50].

Interestingly, up-regulation of miR-34 family of miRNAs, which was also increased in our study, also leads to down-regulation of key targets involved in the cell cycle inducing a senescent phenotype in alveolar epithelial cells of IPF lungs [51], possibly driven by p53 activation [52]. Furthermore, p53 signaling pathway is also affected by miR-502-3p another upregulated miRNA in ILA.

Taken together, our findings indicate that dysregulation of relevant bio-pathological processes such as inflammation, fibrosis and cell senescence may contribute to the development of ILA.

On the other hand, ILA-associated circulating miRNAs could also play a potential role as non-invasive biomarkers for diagnosis. Actually, circulating miRNAs in peripheral biofluids have been widely examined as biomarkers for early diagnosis and monitoring disease progression in many pathological conditions [53,54,55].

Here, we determined the discriminative ability of our upregulated serum miRNAs for identifying ILA subjects from healthy individuals by using ROC curves analysis and demonstrated that levels of miR-193a-5p may have an appropriate diagnostic value as a biomarker, showing an area under the curve of 0.75, with 61% sensitivity at a fixed specificity of 81%.

We acknowledge several limitations of our study. First, the study sample size was relatively small and obtained from a single center. Second, precise mechanisms of how these serum miRNAs function in the lungs to enhance the development of interstitial lung abnormalities are still unclear. Future studies are necessary to identify the actual targets regulated by the discovered miRNAs and their biological function in order to get actual experimental evidence of the mechanistic processes involved in ILA.

In conclusion, in this study we found that respiratory asymptomatic individuals with interstitial lung abnormalities present an over-expression of seven circulatory microRNAs that may affect important pathways, such as inflammation, fibrosis and senescence, which potentially contribute to the pathogenesis of this disorder, and eventually some of them might serve as peripheral biomarkers for screening or auxiliary diagnosis method.

## Figures and Tables

**Table 1:** Demographic and clinical characteristics of (ILA) and Ctrl from the validation cohort.

Characteristics	Ctrl (*n* = 88)	ILA (*n* = 57)	*p*-Value
Gender, male (%)	21 (24)	25 (44)	0.01
Age, yr, mean (SD)	65 (5)	73 (8)	<0.0001
BMI, kg/m2, mean (SD)	27 (5)	26 (4)	0.13
Former smoker, (%)	42 (48)	27 (47)	0.5
Diabetes mellitus, (%)	20 (23)	23(40)	0.02
Hypertension, (%)	29(33)	11(19)	0.08
Gastroesophageal reflux, (%)	39 (44)	22(39)	0.6
Meters W6MT, mean (SD)	450 (101)	408 (154)	0.04
spO2 at rest, mean (SD)	95 (2)	94 (2)	0.03
spO2 post exercise, mean (SD)	92 (5)	88 (9)	0.0004
DL_CO_ adjusted (% predicted) mean (SD)	104 (20)	88 (19)	<0.0001
DL_CO_/VA, mean (SD)	6 (2)	5 (1)	0.001

Yr, year; BMI, body mass index; spO2, oxygen saturation; DL_CO_, diffusion capacity of the lung for carbon monoxide; VA, alveolar; SD, standard deviation. W6MT walking 6 min test. ILA, subjects with interstitial lung abnormalities; Ctrl, control group.

**Table 2:** Diagnostic accuracy values of miR-193a-5p and miR-502-3p as a single and combined biomarker for predicting the presence of ILA in asymptomatic subjects.

MicroRNA	AUC	95% CI	Specificity (%)	Sensitivity (%)	*p*-Value
miR-193a-5p	0.75	0.666–0.831	81	61	< 0.0001
miR-502-5p	0.71	0.622–0.796	81	51	< 0.0001
miR-193a-5p + miR-502-5p	0.75	0.665–0.829	81	51	< 0.0001
ILA: interstitial lung abnormalities. AUC: area under the curve. CI: Confidence interval.					

## Supplementary Materials

The following are available online at https://www.mdpi.com/2073-4409/9/6/1556/s1, Supplementary Figure S1 shows a workflow graph of the study design. Supplementary Figure S2. Levels of miR-16-5p among ILA patients with HRCT predominant pattern of ground glass attenuation and reticular opacities. Graph shows the levels of miR-16-5p in serum of ILA patients analyzed by RT-qPCR. Supplementary Figure S3. Significantly enriched pathways of miR-193a-5p and miR-502-3p. KEGG enriched pathways (non-adjusted p < 0.05) using the experimentally validated targets of ILA-associated miRNAs. Colors indicate p-values and circle size indicates number of target genes in each pathway. Supplementary Table S1. Demographic and clinical characteristics of ILA and Ctrl from the screening cohort Supplementary Table S2. Identification of miRNAs differentially expressed in ILA compared to Ctrl from the screening cohort Supplementary Table S3. Significantly enriched pathways of the seven ILA-associated miRNAs. Experimentally validated targets of five ILA-associated miRNAs were obtained and enriched pathways analysis was performed using the KEGG database and an adjusted p<0.05 (miR-200c-3p, miR-16-5p, miR-21-5p, miR-126-3p and miR-34a-5p). For miR-193a-5p and miR-502-3p, we performed enriched pathway analysis using the KEGG database and a non-adjusted *p* < 0.05. Supplementary Table S4. Correlations between clinical parameters and candidate miRNAs in validation cohort.
