# Supplementary material for: Circulating microRNA Signature Associated to Interstitial Lung Abnormalities in Respiratory Asymptomatic Subjects
Source: Cells. 2020 Jun 26;9(6):1556. doi: 10.3390/cells9061556 (PMC7348836; doi:10.3390/cells9061556)
Supplement: Supplementary file 1 [file cells-09-01556-s001.zip › Supplem figs & Tables/Supplementary Table S4.docx]

| **Supplementary Table S4**. Correlations between clinical parameters and candidate miRNAs in validation cohort. | | | | | | | |
| --- | --- | --- | --- | --- | --- | --- | --- |
| Variable | miR-193a-5p Rho (p value) | miR-532-5p Rho (p value) | miR-200c-3p  Rho (p value) | miR-16-5p  Rho (p value) | miR-21-5p  Rho (p value) | miR-126-3p  Rho (p value) | miR-34a-5p  Rho (p value) |
| Age | 0.05 (0.4) | **0.17 (0.03)** | **0.18 (0.02)** | 0.009 (0.9) | 0.02 (0.8) | 0.11 (0.2) | 0.04 (0.6) |
| BMI | 0.01 (0.8) | -0.03 (0.6) | -0.12 (0.1) | -0.009 (0.9) | -0.11 (0.2) | -0.10 (0.2) | 0.004 (0.9) |
| FVC  (%predicted) | -0.03 (0.6) | 0.08 (0.2) | -0.05 (0.4) | -0.007 (0.9) | -0.04 (0.6) | -0.08 (0.3) | -0.01 (0.8) |
| FEV1  (%predicted) | 0.02 (0.8) | 0.14 (0.08) | -0.03 (0.6) | 0.06 (0.4) | -0.001 (0.9) | -0.007 (0.9) | -0.08 (0.3) |
| DL_CO_ adjusted (%predicted) | **-0.1 (0.04)** | -0.93 (0.7) | -0.06 (0.4) | -0.001 (0.9) | -0.09 (0.3) | -0.008 (0.9) | -0.01 (0.8) |
| DLCO/VA | -0.1 (0.1) | -0.13 (0.09) | -0.09 (0.2) | 0.01 (0.8) | -0.03 (0.7) | 0.03 (0.6) | -0.05 (0.5) |
| SpO2 at rest (%) | -0.01 (0.8) | -0.12 (0.1) | -0.04 (0.5) | -0.03 (0.7) | 0.01 (0.8) | -0.003 (0.9) | -0.03 (0.6) |
| SpO2 post  exercise (%) | -0.07 (0.4) | -0.10 (0.2) | **-0.20 (0.01)** | -0.1 (0.14) | -0.13 (0.1) | **-0.16 (0.05)** | -0.06 (0.4) |
| Meters W6MT | -0.04 (0.6) | -0.10 (0.2) | **-0.16 (0.05)** | -0.07 (0.3) | 0.06 (0.4) | -0.02 (0.8) | **-0.2 (0.01)** |
| BMI, body mass index; spO2, Oxygen saturation; DL_CO_, monoxide diffusing capacity; VA, volume alveolar; W6MT walking 6 minutes test. | | | | | | | |
