# Supplementary material for: Circulating microRNA Signature Associated to Interstitial Lung Abnormalities in Respiratory Asymptomatic Subjects
Source: Cells. 2020 Jun 26;9(6):1556. doi: 10.3390/cells9061556 (PMC7348836; doi:10.3390/cells9061556)
Supplement: Supplementary file 1 [file cells-09-01556-s001.zip › Supplem figs & Tables/Supplementary Table S2.docx]

| **Supplementary Table S2**. Identification of miRNAs differentially expressed in ILA compared to Ctrl from the screening cohort | | |
| --- | --- | --- |
| **MicroRNA** | **p-value** | **Log2 FoldChange** |
| miR-532-5p | 0.017 | 2.74 |
| miR-193a-5p | 0.018 | 2.3 |
| miR-16-5p | 0.028 | 2.14 |
| miR-744 | 0.049 | 2.11 |
| miR-95-3p | 0.029 | -1.42 |
| miR-200c-3p | 0.01 | -1.48 |
| miR-502-3p | 0.032 | -1.49 |
| miR-9-5p | 0.04 | -1.54 |
