# Supplementary material for: Circulating microRNA Signature Associated to Interstitial Lung Abnormalities in Respiratory Asymptomatic Subjects
Source: Cells. 2020 Jun 26;9(6):1556. doi: 10.3390/cells9061556 (PMC7348836; doi:10.3390/cells9061556)
Supplement: Supplementary file 1 [file cells-09-01556-s001.zip › Supplem figs & Tables/Supplementary Table S1.docx]

| **Supplementary Table S1**. Demographic and clinical characteristics of ILA and Ctrl from the screening cohort | | | |
| --- | --- | --- | --- |
| **Characteristics** | **Ctrl**  **(n=24)** | **ILA**  **(n=24)** | **p-value** |
| Gender, male (%) | 11 (46) | 12 (50) | 0.5 |
| Age, yr mean (SD) | 65 + 6 | 71+ 6 | 0.001 |
| BMI, (SD) | 28 + 5 | 27 + 3 | 0.3 |
| Former smoker, (%) | 5(21) | 12 (50) | 0.06 |
| Diabetes mellitus, (%) | 6 (25) | 4(17) | 0.7 |
| Hypertension, (%) | 7 (29) | 8 (33) | 0.5 |
| Gastroesophageal reflux, (%) | 15(63) | 14(58) | 0.5 |
| Meters W6MT, mean (SD) | 461 + 100 | 441 + 113 | 0.7 |
| spO2 at rest, mean (SD) | 95 + 2 | 94 + 2 | 0.09 |
| spO2 post exercise, mean (SD) | 93 + 3 | 88 + 8 | 0.003 |
| DL_CO_ adjusted (% predicted), mean (SD) | 111 + 20 | 85 + 21 | <0.0001 |
| DL_CO_/VA, mean (SD) | 6 + 1 | 5 + 1 | <0.0001 |
| Yr, year; BMI, body mass index; spO2, Oxygen saturation; DL_CO_, monoxide diffusing capacity; VA, volume alveolar; SD, standard deviation. W6MT walking 6 minutes test. | | | |
