# Supplementary figures and images for: Circulating microRNA Signature Associated to Interstitial Lung Abnormalities in Respiratory Asymptomatic Subjects
Source: Cells. 2020 Jun 26;9(6):1556. doi: 10.3390/cells9061556 (PMC7348836; doi:10.3390/cells9061556)

# miR-16-5p

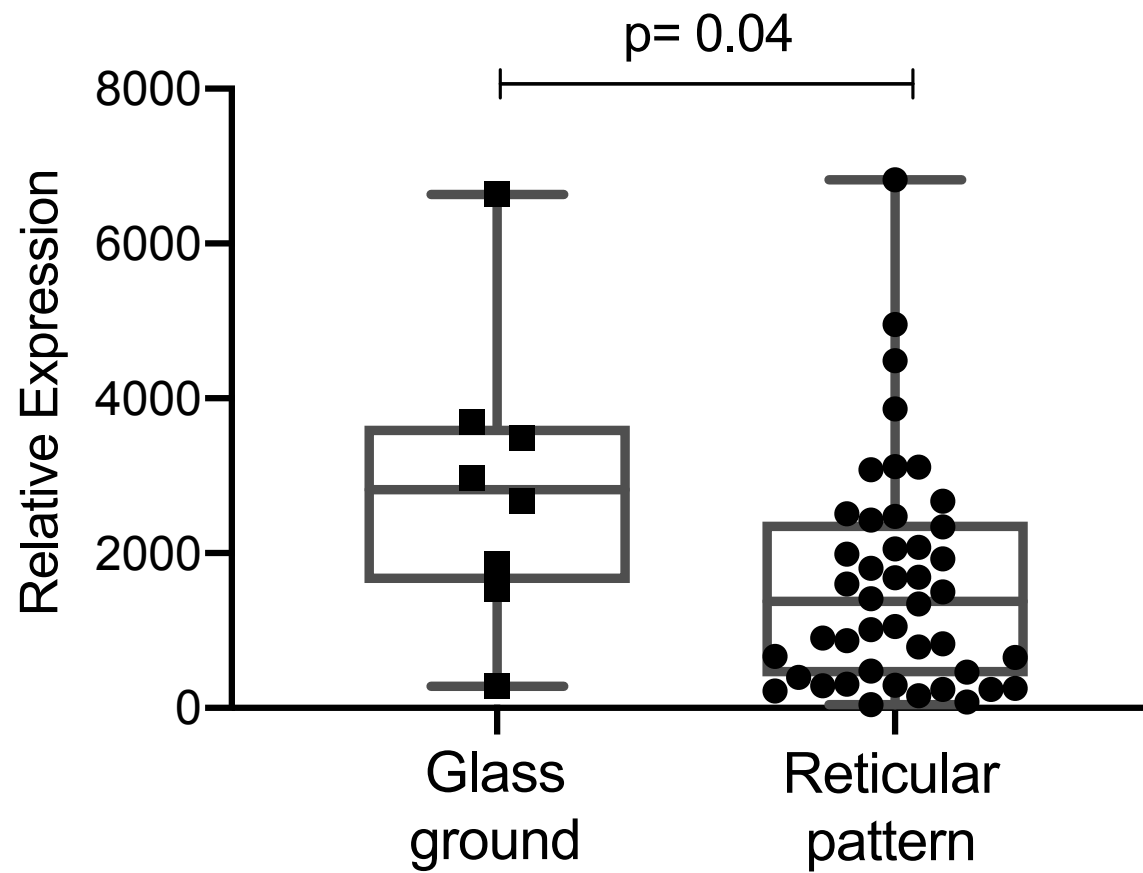

Supplement: Supplementary file 1 [file cells-09-01556-s001.zip › Supplem figs & Tables/Suppl Fig S2.pdf]

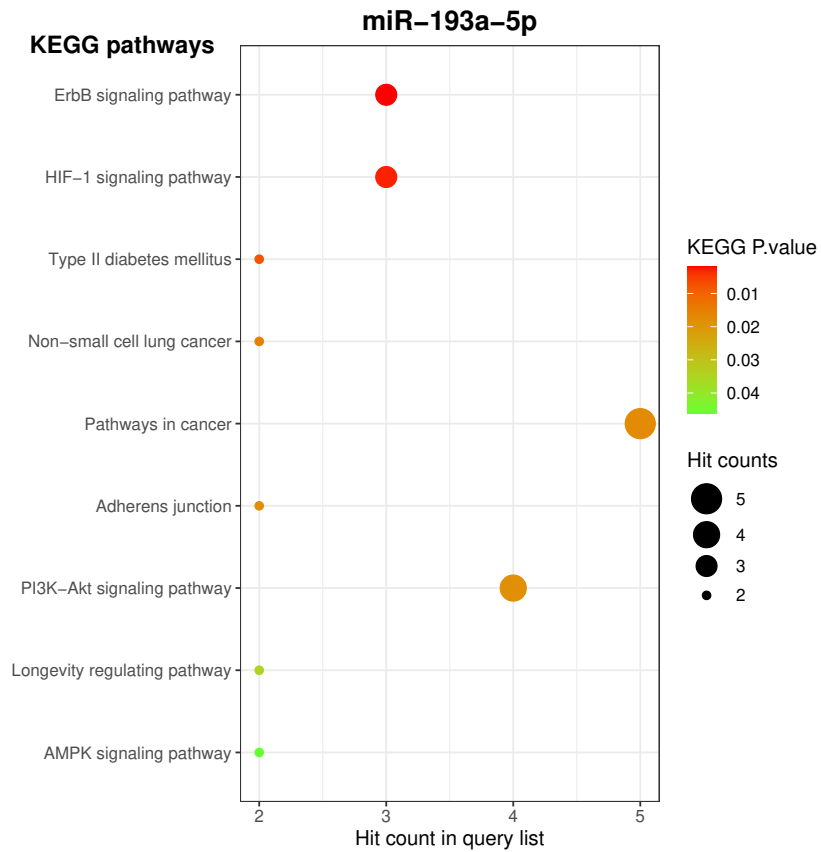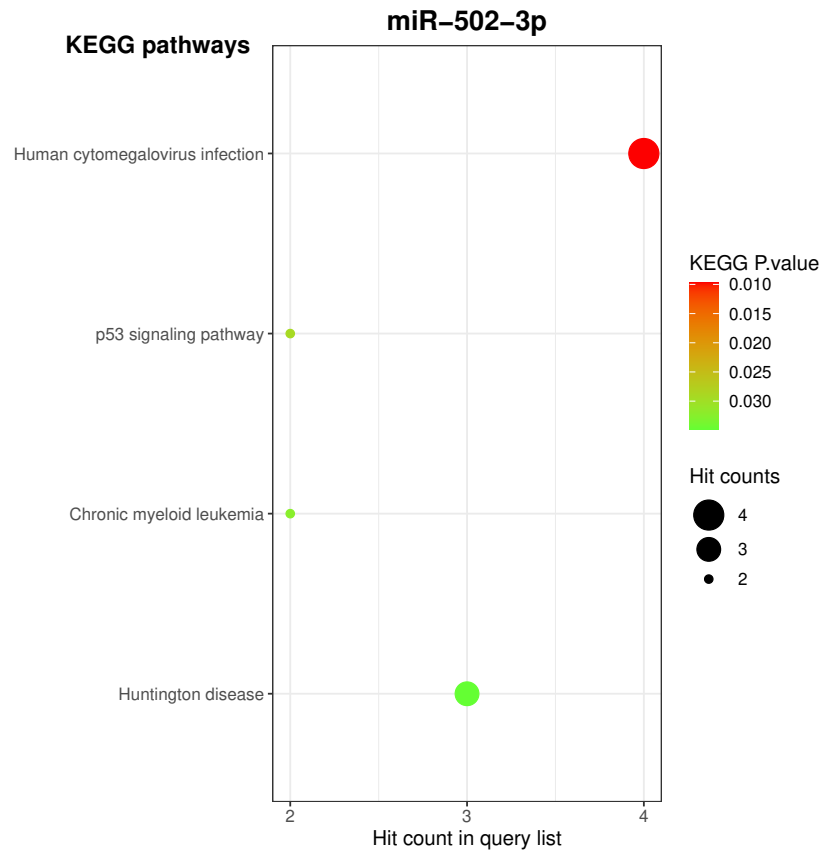

Supplement: Supplementary file 1 [file cells-09-01556-s001.zip › Supplem figs & Tables/Suppl Fig S3.pdf]

# Study Workflow

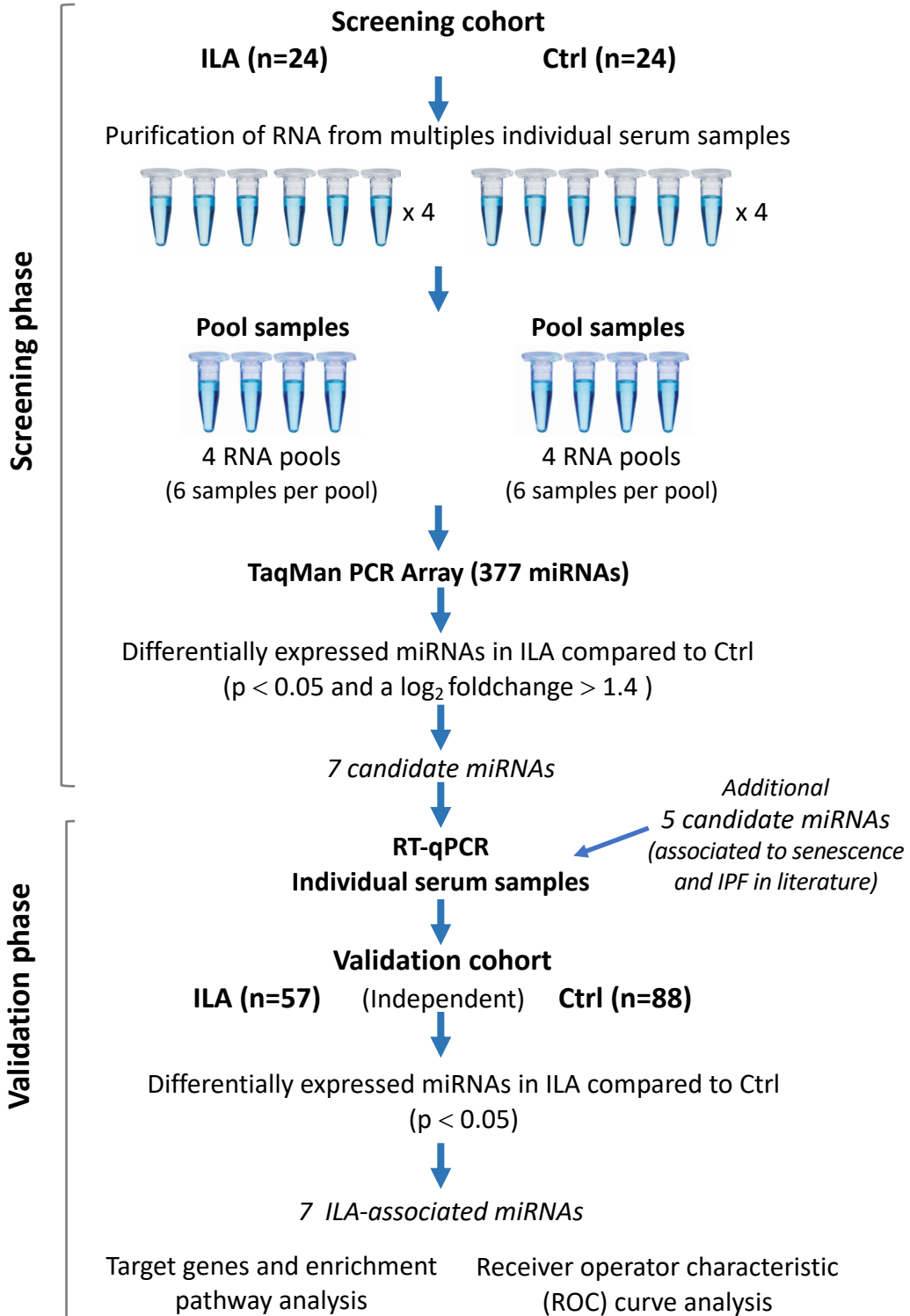

Supplement: Supplementary file 1 [file cells-09-01556-s001.zip › Supplem figs & Tables/Suppl Fig S1 study workflow.pdf]
